# The Emerging Role of Human Gut Bacteria Extracellular Vesicles in Mental Disorders and Developing New Pharmaceuticals

**DOI:** 10.3390/cimb46050286

**Published:** 2024-05-15

**Authors:** Effrosyni Louka, Vassiliki Lila Koumandou

**Affiliations:** Genetics Laboratory, Department of Biotechnology, Agricultural University of Athens, Iera Odos 75, 11855 Athens, Greece; elouka@aua.gr

**Keywords:** extracellular vesicles, intestinal microbiota, gut–brain axis, mental disorders

## Abstract

In recent years, further evidence has emerged regarding the involvement of extracellular vesicles in various human physiopathological conditions such as Alzheimer’s disease, Parkinson’s disease, irritable bowel syndrome, and mental disorders. The biogenesis and cargo of such vesicles may reveal their impact on human health nd disease and set the underpinnings for the development of novel chemical compounds and pharmaceuticals. In this review, we examine the link between bacteria-derived exosomes in the gastrointestinal tract and mental disorders, such as depression and anxiety disorders. Crucially, we focus on whether changes in the gut environment affect the human mental state or the other way around. Furthermore, the possibility of handling bacteria-derived exosomes as vectors of chemicals to treat such conditions is examined.

## 1. Introduction

Extracellular vesicles (EVs) are membrane nanoscale vesicles naturally secreted by cells into the extracellular space. They play an important role in intercellular communication in numerous physiological and pathological processes. EVs can be classified based on their cellular origin, biogenesis, and/or biological function [1]. Based on their biogenesis, the three major classes of EVs are exosomes, microvesicles, and apoptotic bodies [2,3]. Exosomes, also referred to as intraluminal vesicles (ILVs), are a subtype of EV of endocytic origin, typically 30–150 nm in diameter, enclosed by a single outer membrane, and secreted by all eukaryotic cell types; they have been found in plasma, serum, saliva, urine, semen, bronchial fluid, cerebrospinal fluid (CSF), breast milk, amniotic fluid, synovial fluid, tears, lymph, bile, and gastric acid [4]. Since the EV field has grown, different types of vesicles have been described; a recent study suggests a more specialized classification, adding autophagic EVs, stressed EVs, exomeres, non-vesicular particles, and matrix vesicles to the existing ones [5]. Notably, existing technologies cannot distinguish the subtypes well, and no clear consensus has been reached on the specific markers of the subtypes of EVs [6]. EVs are referred to by various names depending on the organism from which they originate, including OMVs (outer membrane vesicles) in Gram-negative bacteria and EVs or MVs (extracellular vesicles or membrane vesicles) in Gram-positive bacteria [7]. To ensure clarity and precision in scientific communication, we quote the International Society for Extracellular Vesicles’ use of “extracellular vesicle” (EV) as the universal term for particles naturally discharged from cells, enclosed by a lipid bilayer, and lacking the ability to replicate, meaning they lack a functional nucleus [2].

EVs contain proteins, lipids, nucleic acids (including DNA, mRNA, and non-coding RNA), and small metabolites [8]. Through this cargo, EVs can mediate cell communication, modulating downstream signaling pathways in recipient cells [9]. Due to their diverse array of bioactive proteins, adhesion molecules, and membrane-anchored receptors, EVs are well suited for tailored communication with their surroundings. Through fusion with target cells, EVs can convey their original cytosolic contents and relocate both membrane-attached and membrane-spanning proteins [10]. EVs are involved in interactions between pathogenic microorganisms and their animal or plant hosts [11]. Exosomes in eukaryotic cells are enriched with small RNAs and play a role in many physiological processes [1]. Due to the impressive ability of EVs to package and maintain the molecular characteristics of their originating cells, they have become promising resources for uncovering biomarkers [12].

Bacteria (pathogenic and non-pathogenic species) release EVs, which range in size from 10 to 300 nm and contain outer membrane proteins, periplasmic proteins, lipoproteins, phospholipids, and lipopolysaccharides (LPS), as well as cytoplasmic proteins and DNA [13]. The role of prokaryotic EVs lies in pathogenicity, intercellular communication (quorum sensing), and the transfer of genetic material and proteins to other bacteria. In addition, they have the ability to stimulate the host’s immune response, providing a tool for vaccine development [14]. Furthermore, bacterial EVs (BEVs) play critical roles in both inter-bacteria and bacteria–host interactions and are involved in bacterial survival, biofilm formation, horizontal gene transfer, stress response, nutrient acquisition, toxin delivery, and antibiotic resistance [15].

One of the main challenges in studying EVs is a lack of methods to quantify EVs that are sensitive enough to differentiate EVs from similarly sized lipoproteins and protein aggregates [16]. Ultracentrifugation is the gold standard method for EV isolation, including differential ultracentrifugation, density gradient centrifugation, and rate-zonal centrifugation techniques [17]. Lately, the emergence of microfluidic chips, nanolithography, electro-deposition, and other technologies has promoted innovation and combinations of isolation methods [18]. Bacterial EVs in human body fluids have been less thoroughly investigated, likely due to the methodological challenges in separating BEVs from their matrix and host-derived eukaryotic EVs. A method combining ultrafiltration, size-exclusion chromatography, and density gradient centrifugation ensured the integrity of the isolated BEVs and eliminated the need for labeling, facilitating their subsequent characterization [19].

The human gastrointestinal (GI) tract is composed of multiple different organs and can be divided into the upper and lower GI tract. The upper GI tract refers to the mouth, esophagus, stomach duodenum, jejunum, and ileum, while the colon, rectum, and anus make up the lower GI tract. The overall function of the GI tract is to digest ingested nutrients through digestive enzyme secretion and nutrient absorption [20]. The GI tract is colonized by a diverse microbial community—bacteria, fungi, archaea, and viruses—termed the gut microbiota [21], which is essential to intestinal homeostasis and human health. The flora includes large populations of *Lactobacillus*, *Streptococcus*, *Staphylococcus*, Enterobacteriaceae (stomach and duodenum), *Bifidobacterium*, *Bacteroides*, *Lactobacillus*, *Streptococcus*, Enterobacteriaceae (jejunum, and ileum), *Bacteroides*, *Eubacterium*, *Clostridium*, *Peptostreptococcus*, *Streptococcus*, *Bifidobacterium*, *Fusobacterium*, *Lactobacillus*, and Enterobacteriaceae (colon) [22].

The gastrointestinal microbiota play pivotal roles in neurodevelopmental processes and brain functions through the microbiota–gut–brain axis. The dysregulation of this axis by endogenous and exogenous factors, such as aging and stress, accelerates the occurrence of psychiatric disorders [23]. EVs have several advantages over conventional synthetic drug carriers, opening new possibilities for modern drug delivery [24] and alternative approaches for the treatment of psychiatric disorders.

## 2. The Gut–Brain Axis

The microbiota–gut–brain axis refers to wide-ranging interactions between the gut microbiota and the central nervous system (CNS), which involve endocrine, immune, and neural signaling pathways [25]. The gut microbiome, in addition to its contribution to food digestion and nutrient metabolism, plays a fundamental role in host immune system development and modulation of the gut barrier and immune responses [26]. The primary avenues of bidirectional communication include the immune system, the vagus nerve system, the neuroendocrine system, the circulatory system, and the enteric nervous system [27].

A growing number of studies address the contribution of the microbiota–gut–brain axis to neurodevelopment and mental health (Table 1). For example, plasma adrenocorticotropic hormone (ACTH) and corticosterone elevation in response to restraint stress (a method used to induce physiological responses in an animal by restricting its free movement) was substantially higher in germ-free (GF) mice than in specific-pathogen-free (SPF) mice; GF mice also exhibited reduced expression of brain-derived neurotrophic factor (BDNF) in the cortex and hippocampus relative to SPF mice [28]. Many studies indicate that microbiota- and host-derived miRNAs regulate each other: gut bacteria have a great impact on host cell miRNA expression, and host miRNAs shape and regulate the gut microbiota [29,30,31,32]. Dalmasso et al. compared miRNA expression in GF mice and those colonized with microbiota, finding specific miRNAs upregulated in the mucosal tissues of the ileum and colon, while others were downregulated in the colon upon colonization [33]. Moreover, oral administration of miR-30d, also present in EVs, from the feces of multiple sclerosis (MS) patients suppressed MS-like symptoms in mice by expanding *Akkermansia muciniphila* populations [34]. Chronic stress significantly alters the intestinal microbiota composition, primarily depleting Lactobacilli, while ROS produced by Lactobacilli can inhibit kynurenine metabolism, a pathway that can negatively impact the brain when dysregulated [35]. 

The main neurological pathway of the gut–brain–axis (GBA) is the vagus nerve [43]. The gut microbiome has the ability to stimulate the vagus nerve, possibly through the subepithelial region, which could serve as a protected space for interaction between the nerves of the intestinal tract and the gut microbiota, conveying information from the digestive system to the nucleus tractus solitarius, which in turn relays this information to various parts of the central autonomic network via different neuronal routes, resulting in distinct physiological effects [25,44]. Vagal afferent fibers are polymodal and therefore respond to mechanical, chemical, or hormonal signals; they can sense gut bacteria and their metabolites and transfer this information to the CNS [45,46,47]. The detection of gut bacteria and their byproducts is enabled by unique sensory epithelial cells within the gut, which were initially identified in 2015 and are commonly referred to as “neuropods” [48].

The immune system also serves as a major communication pathway in the microbiota–gut–brain axis [49]. In a healthy state, the gut employs various defense mechanisms to maintain balance and prevent pathogen invasion (eubiosis). The mucus layer acts as a barrier, while dendritic cells sample bacteria within the mucosal surface, presenting antigens to immune cells in the mesenteric lymph node. Here, molecules like IgA and antimicrobial peptides inhibit pathogen spread, and regulatory T cells are induced, promoting tolerance to dietary and commensal antigens. In contrast, disruption of the microbiome (dysbiosis) triggers an innate immune response, with pathogens and toxins (also released by BEVs) recognized by pattern recognition receptors, leading to inflammation and increased gut permeability. These processes can result in the systemic circulation of inflammatory mediators, impacting the CNS [50]. 

Gut–brain axis communication involves several neurotransmitters, including serotonin, dopamine, noradrenaline, and gamma-aminobutyric acid (GABA), synthesized not only in the CNS but also in enteroendocrine cells influenced by intestinal peptides and the gut microbiota (Table 2). Serotonin, predominantly synthesized by enterochromaffin cells (ECCs), regulates intestinal reflexes, mediates gut-brain communication, and affects the immune system [51,52]. Certain gut microbes can directly produce serotonin, influencing its levels and activity, which in turn impacts gut microbiota composition [53,54]. Similarly, dopamine and norepinephrine, synthesized by both gut microbes and the CNS, have a role in gut-brain communication [53]. The gut microbiota can synthesize oxidases that affect catecholamine metabolism and transport, influencing neurotransmitter availability [55]. Additionally, GABA, synthesized by intestinal bacteria such as *Bifidobacterium* and *Lactobacillus*, affects brain function. GABA serves as the primary inhibitory neurotransmitter in the CNS and is widely distributed throughout the brain, participating in approximately 40% of inhibitory synapses in adult vertebrates. Synthesized within the CNS through the decarboxylation of glutamic acid, GABA exerts its inhibitory actions via two distinct receptor types: GABAa (ionotropic) and GABAb (metabotropic) [56]. There is ample evidence indicating the crucial involvement of GABA in controlling stress levels in the brain, a key factor contributing to vulnerability in mood disorders [57]. Sanacora et.al. demonstrated a significant (52%) reduction in the occipital cortex GABA levels of depressed patients compared with healthy subjects [58]. Lactate, a substrate for short-chain fatty acids (SCFAs) produced by gut microbes, modulates GABA transmission via specific receptors, GABAb [59], while probiotic administration alters GABAB1b/GABAAα2 [60] receptor expression, affecting behavior. The concentration of GABA in human plasma has been thoroughly investigated, especially in studies related to psychiatry. However, the findings thus far have been inconclusive, likely because of variations in the analytical techniques employed across different studies. According to a study by Song et al., the plasma GABA levels in healthy subjects were found to be 98.6 ± 33.9 ng/mL [61] (approximately equal to 0.96 mM). On the contrary, an in-depth screening of human intestinal *Bacteroides* showed that most strains tested produced GABA at concentrations ranging from 0.09 to 60.84 mM. Those findings indicate the protective mechanism of GABA against acid stress in *Bacteroides* and, altogether, suggest an important contribution of *Bacteroides* in the regulation of the GABAergic system in the human gut [62]. These outcomes underscore the complex interplay between gut microbiota-derived neurotransmitters and host physiology, with implications for brain health and behavior [50].

## 3. EVs and Mental Disorders

According to the World Health Organization, a mental disorder, or a mental health condition, is characterized by a clinically significant disturbance in an individual’s cognition, emotional regulation, or behavior. It is usually associated with distress or impairment in important areas of functioning. The most common types of mental disorders include neurodevelopmental disorders, anxiety disorders, depression, bipolar disorder, post-traumatic stress disorder, schizophrenia, eating disorders, disruptive behavior, and dissocial disorders. The broader term “Neurodevelopmental disorders” covers mental disorders, psychosocial disabilities, and other mental states associated with significant distress, impairment in functioning, or the risk of self-harm. A total of 970 million people around the world were living with a mental disorder in 2019 (one in every eight people), with anxiety and depressive disorders being the most common. In 2020, because of the COVID-19 pandemic, cases of anxiety rose by an estimated 26% and cases of major depressive disorders rose by 28% in just one year [76].

Intestinal balance relies on intricate and ever-changing relationships among the microbiota, the epithelium, and the host immune system. Due to the intricate nature of the intestinal environment, various regulatory mechanisms, such as immune receptors, signaling pathways, regulatory proteins, and miRNAs, are necessary to maintain harmony and prevent microbial imbalances, also known as dysbiosis. Intercellular communication plays a pivotal role in coordinating appropriate responses to uphold intestinal equilibrium. Increasing evidence suggests that communication within and between different kingdoms is facilitated by EVs released by either the gut microbiota or host intestinal cells [32]. While gut EVs themselves do not have the ability to directly cause or treat diseases, they can indirectly influence disease by imparting both harmful and beneficial signals [77]. Dysbiosis within the gut flora has been linked to various systemic conditions, including functional bowel disorders [78], inflammatory diseases [79], atherosclerosis [80], metabolic disorders [81], and neuropsychiatric conditions [82].

In humans, Alzheimer’s disease [83], Parkinson’s disease [84], autism [85], irritable bowel syndrome [86], depression, and anxiety [87] all involve alterations of the microbiome, but a direct causal role for the altered microbial composition observed in these diseases has yet to be confirmed. Despite conflicting results, a growing body of research on schizophrenia, major depressive disorder (MDD), bipolar disorder, substance abuse, and post-traumatic stress disorder has provided initial indications of dysregulation in EV-derived miRNAs identified in serum or plasma samples [88]. When GF rodents received fecal microbiota transplants from MDD patients, they exhibited symptoms resembling depression, whereas transplants from healthy individuals did not induce similar behavior [89,90]. However, the relationship between MDD and dysregulation of the gut microbiota is complex and not fully understood; it is challenging to determine a definitive sequence of events because the relationship likely involves bidirectional influences. EVs from both the host and intestinal microorganisms collectively influence the function of the intestinal mucosal barrier and play a crucial role in bacterial–host communication [91,92]. Dysfunction in the gut–brain axis is a hallmark of MDD, potentially leading to underlying inflammation, imbalance in the hypothalamic–pituitary axis, and alterations in neural, metabolic, and endocrine pathways. Studies in mice have shown that EVs derived from *Lactobacillus* can modulate the expression of BDNF in the hippocampus, producing antidepressant-like effects in stress-induced depression model mice. Additionally, EVs from the intestinal flora of mice can impact the expression of inflammatory markers and regulate serotonin signaling and metabolism via the GBA [6]. Depression and anxiety may be associated with persistent neuroinflammation. Neuroinflammation can be triggered by alterations in the microbiome, heightened intestinal permeability, and/or elevated serum levels of LPS; thus, disruptions in the gut microbiota due to external stresses can lead to inflammation in the gastrointestinal tract, which in turn may lead to memory deficits [93]. BEVs with LPS cargo have been detected in the plasma of patients with intestinal barrier dysfunction, capable of triggering immune activation, and are associated with compromised barrier function in individuals diagnosed with inflammatory bowel disease, HIV, and intestinal mucositis induced by cancer therapy [19]. Intestinal bacteria produce EVs containing antigens that can activate Toll-like receptors (TLRs) on epithelial cells or immune cells [94]. TLR4 is specifically stimulated by LPS and has garnered significant attention in depression research [95,96]. Moreover, EVs from both bacteria and host cells have been demonstrated to augment blood–brain barrier (BBB) permeability, facilitating the entry of bacterial products into the brain and contributing to neuroinflammation in Parkinson’s disease and meningitis [36,97]. 

Research into the potential role of gut bacteria in crossing the BBB and causing neuroinflammation is still an area of active investigation. While there is evidence to suggest that gut bacteria can influence brain function through various pathways [98], the specific mechanisms by which gut bacteria might directly cross the BBB and induce neuroinflammation remain less clear. Furthermore, various circulating EV miRNAs, known to predict behavioral changes induced by chronic social defeat stress, have been found to regulate the production of pro-inflammatory cytokines like IL-1β, TNF-α, and IL-6 in mouse models [99]. Bacterial EVs have been observed to possess the ability to penetrate the BBB, yet the precise mechanisms facilitating this crossing are not well understood. Moreover, there is a notable scarcity of data regarding EVs released by the human gut microbiota in this specific context [100]. Tumor necrosis factor α (TNF-α) serves as an essential mediator in the stress response of mice, with it being crucial for stress-induced synaptic potentiation in the ventral hippocampus and for the elevation of anxiety-like behavior [101]. Thus, using TNF-α as a potential novel therapeutic target and utilizing EVs as vectors may represent a promising novel therapeutic strategy for stress-related disorders. However, there are unresolved questions in this area that require additional research and exploration.

The complex communication between the gut microbiota and the brain plays a crucial role in the development of neurodegenerative disorders such as Alzheimer’s disease (AD). Changes in the GBA can profoundly impact disease progression through various mechanisms, including heightened permeability of the gastrointestinal barrier and excessive immune activation, which triggers systemic inflammation. This inflammation can compromise the integrity of the BBB, facilitating neural damage, neuroinflammation, and, ultimately, neurodegeneration [98,102]. Parkinson’s disease (PD) is a predominant movement disorder and ranks as the second most prevalent neurodegenerative condition following AD. The disease’s pathology is believed to initiate in the enteric nervous system before progressing to the brain through the vagus nerve [84]. The bacteria found in the gut microbiome along with their byproducts, including LPS, have been suggested as potential contributors to both PD and AD, with the activation of microglia playing a crucial role in the disease’s progression. Bacteria inhabiting the gut microbiota can secrete LPS and amyloids. These compounds have the potential to activate microglia in the brain, leading to the release of proinflammatory cytokines, which are implicated in the development of AD [103,104]. *Escherichia coli*, *Salmonella enterica*, *Salmonella typhimurium*, *Bacillus subtilis*, *Mycobacterium tuberculosis*, and *Staphylococcus aureus* are some of the many bacterial species that produce functional extracellular amyloid fibers [105]. Curli amyloid and its homologs, derived from enteric biofilms, have been connected to neurodegeneration and autoimmunity; nevertheless, the specific conditions under which bacterial amyloids impact these diseases need further examination [106].

Anxiety and depression frequently manifest as symptoms in patients with PD and are recognized as risk factors for the development of dementia and AD [107,108]. The potential biological pathways connecting depression to dementia encompass vascular disease, changes in glucocorticoid steroids and hippocampal atrophy, heightened accumulation of β-amyloid plaques, inflammatory processes, and deficiencies in nerve growth factors [109]. The precise mechanisms underlying the connections between anxiety disorders and dementia remain unclear, but they may involve various pathways, such as changes in the hypothalamic–pituitary–adrenal (HPA) axis, as well as lifestyle and psychosocial factors like diet, social support, and exercise. Additionally, the mechanisms might vary depending on the specific type of anxiety disorder [110].

Hence, the correlation between anxiety, depression, and dementia could potentially be elucidated through a shared mechanism wherein harmful EVs released by gut microbiome bacteria traverse the brain, precipitating a range of neurological disorders. The manifestation of these disorders may vary depending on the specific profile of EVs and/or individual genetic and environmental influences [111].

## 4. EVs and Antidepressant Treatment

There is increasing evidence indicating that the EVs produced by commensal bacteria play a crucial role in communication between the microbiome, the gut, and the brain. EVs possess the advantage of better access to the bloodstream than entire microbes, enabling them to travel to the CNS. Consequently, EVs aid in delivering concentrated signaling molecules and fragile cargo, like RNA, which would be vulnerable if transported from the gut to the brain without protection [112]. Within the CNS, the blood–brain barrier impedes the desired therapeutic outcomes because of challenges in effectively targeting, controlling release timing, and attaining adequate therapeutic levels in the brain. Consequently, the majority of potentially beneficial diagnostic and therapeutic substances cannot access the brain when administered systemically.

EVs are emerging as a promising tool for therapeutic delivery owing to their favorable intrinsic features of biocompatibility, stability, stealth capacity, ability to overcome natural barriers, and inherent homing capability [113]. The lipid membranes of EVs and BEVs shield cargo from the immune system of the host, while surface ligands on the membrane enable precise targeting of specific cell types over long distances [114]. Sorting cargo to EVs is likely a controlled procedure, not occurring randomly, and EVs serve critical functions in facilitating cell-to-cell communication. Encapsulation within EVs offers shielding against both the enzymatic and non-enzymatic degradation of cargo, while also enabling cellular uptake through endocytosis [115]. A beneficial role of intestinal epithelial cell-derived EVs was observed when they activated neuron growth via their sRNA cargo. Intestinal cells treated with carnosine, contained abundantly in chicken breast meat, significantly induced neurite growth [116]. One significant benefit of compartmentalized BEV-mediated cargo transportation is the prevention of signal molecule dilution during extended journeys. This facilitates the effective delivery of the molecule to target cells, ensuring that concentrations surpass the threshold required to trigger desired effects. Additionally, EVs released by the microbiome possess the ability to enter the bloodstream, traverse the BBB, and contribute to hallmark pathological effects observed in neurological disorders, such as Alzheimer’s disease, including tau phosphorylation, neuroinflammation, and cognitive impairments [111]. EVs, particularly those containing specific circular RNAs or microRNAs (miRNAs), hold promise as potential therapeutic agents for depression and other CNS diseases. Targeting EVs carrying circDYM (circular RNA DYM) to the brain has been shown to reduce depression-like behavior induced by chronic unpredictable stress. These EVs inhibit microglial activation, maintain BBB integrity, reduce peripheral immune cell infiltration, and alleviate astrocyte dysfunction. Additionally, EVs derived from natural killer cells have demonstrated the ability to pass through the BBB, target astrocytes, and reduce depressive behavior when containing specific miRNAs such as miR-207 [117,118].

EVs from *Lactobacillus rhamnosus* JB-1 have the ability to stimulate primary afferent vagal neurons in the intestines and alleviate behaviors resembling anxiety and depression in mice, increasing specific GABA receptor levels, crucial for mood and anxiety disorders [60]. BEVs from *L. rhamnosus* JB-1 have potent immunoregulatory effects as they replicate the ability of whole bacteria to decrease the amplitude of nerve-dependent colon migrating motor complexes [41]. These effects occur rapidly and only when BEVs are placed on the intestinal epithelium, not on neurons directly [111]. Furthermore, CD4 + CD25+ regulatory T cells have been shown to mediate at least some of the anxiolytic- and antidepressant-like effects of the psychoactive bacteria *L. rhamnosus* JB-1 [119]. Notably, SCFAs such as acetate, propionate, and butyrate, are believed to play a crucial role in the effects of the gut microbiota on the host. SCFAs are produced in the gut through the bacterial fermentation of dietary fiber, and emerging evidence suggests that SCFAs influence human psychobiology through various pathways including endocrine, neural, and immune. Data have illustrated that SCFAs act as mediators in microbiota–gut–brain interactions and impact the acute stress response, eating behavior, and nutritional status in malnourished patients with anorexia nervosa [120]. Additionally, they have a pivotal function in cognitive function and pathology in AD and PD. SCFAs impact the microglia transcriptome, and immune cell recruitment possibly promotes glutamate–glutamine shuttle to potentially resist oxidative damage in neurons at the cellular level. The findings imply that specialized diets (supplemented with high acetate and butyrate) releasing high amounts of SCFAs may have a neuroprotective effect [77,121]. Gut bacteria possess neurotransmitters that can affect brain function and behavior, and they have the ability to encapsulate and release these molecules within EVs, allowing for their protection and efficient transport throughout the body at heightened concentrations. Changes in neurotransmitter levels have been linked to various neurological disorders (such as proposed deficits in serotonin and GABA in major depression [122], and reduced dopamine production in PD [123]). However, the potential impact of neurotransmitter-carrying bacterial EVs on these conditions remains unexplored.

Probiotics have been shown to be effective against infectious diseases in clinical trials, with either intestinal or extraintestinal health benefits. Even though probiotic effects are strain specific, some “widespread effects” include pathogen inhibition, the enhancement of barrier integrity, and the regulation of immune responses. The mechanisms involved in the health benefits of probiotics are not completely understood, but these effects can be mediated, at least in part, by probiotic-derived EVs [124]. Increasingly, research is focussing on searching for alternatives to probiotics, such as postbiotics, a mixture of metabolic products or non-viable fragments of probiotics that have a beneficial effect on the functioning of the human body [125]. For example, EVs from the probiotic *Lactobacillus plantarum* (*L*-EVs) can change the expression of neurotrophic factors in the hippocampus and afford antidepressant-like effects in mice with stress-induced depression [38]. These data indicate that the antidepressant-like effects of *L*-EVs are comparable to those of imipramine [38], a typical tricyclic antidepressant. Regrettably, the existing therapeutics presently accessible fall short; their inefficacy, adverse effects, and associated risks leave patients with few treatment alternatives [126]. Given the minimum 30% rate of treatment-resistant depression [127], among different countries, discovering novel treatment pathways for patients with depression is more than crucial.

The observed positive impact of both intestinal epithelial cell-derived EVs and microbiome-derived EVs on promoting neuron growth through their small RNA cargo underscores the importance of dietary interventions, particularly focusing on incorporating components like carnosine. Thus, emphasizing dietary considerations could be crucial in the treatment and management of neurological conditions.

The administration of *Bacteroides fragilis*, a Gram-negative anaerobe that colonizes the GI tract of mammals, significantly reduced the development of intestinal inflammation and damage in experimental models of colitis in mice [128]. EVs derived from *Escherichia coli* Nissle 1917 were able to mediate the anti-inflammatory and barrier protective effects in a dextran sulfate sodium-induced colitis mouse model [91]. According to the inflammatory theory of depression, it is suggested that inflammation in the body plays a role in the development of depression [129,130]. It would be of great scientific interest to examine the therapeutic potential of targeting the immune system, utilizing engineered EVs, to treat depression.

Drug-loaded exosomes have been used in PD treatment (Table 3). Blood exosomes were loaded with dopamine and successfully delivered dopamine to the brain as the brain distribution of dopamine increased >15-fold. Exosomes carrying dopamine exhibited superior therapeutic effectiveness in a mouse model of PD and demonstrated reduced systemic toxicity, compared to freely administered dopamine via intravenous delivery [131]. Accordingly, EVs containing curcumin protected mice from LPS-induced septic shock, indicating that EVs can enhance the stability, solubility, and bioavailability of curcumin when used as carriers [77]. Another study demonstrated that daily intranasal administration of curcumin-loaded EVs reduced the severity of experimental autoimmune encephalomyelitis, potentially by promoting apoptosis in microglial cells [132].

Emerging evidence indicates the potential of EVs and their contents as biomarkers for monitoring the response to antidepressant treatment in individuals with MDD. Those findings include significant changes in EVs and their contents, before and after antidepressant treatment in patients with MDD, including mitochondrial-related proteins, BDNF/pro-BDNF levels, and certain miRNAs. Specifically, mitochondrial-related protein abnormalities in brain-derived EVs tend to normalize following antidepressant treatment. BDNF levels in patients’ plasma EVs are reduced before treatment but show no significant difference compared to controls after treatment; the anticipated levels of BDNF before and after treatment were not detected, possibly due to undetermined EV sources and peripheral mechanisms. Specific miRNAs, including miR-21-5p, miR-30d-5p, and miR-486-5p, were found to change significantly during antidepressant treatment and were associated with treatment response, as indicated by stepwise regression analysis. Overall, these findings suggest that EVs and their contents may serve as potential biomarkers for monitoring antidepressant treatment response in MDD patients [155,156]. 

There are still numerous challenges to address before EV-based therapeutics can be applied clinically. These include issues like specificity, stability, biodistribution, storage, large-scale production, and the thorough examination of EV composition [157]. Considering the compatibility between the cells of origin and the target cells is of great importance when designing exosome-based therapies [158]. As an illustrative example, using *S. aureus* vesicles for targeted antibiotic delivery offers a tailored and efficient approach to combatting *S. aureus* infections, by leveraging specificity between the source and target and enhanced internalization, thereby enhancing the therapeutic effectiveness of the treatment [149].

## 5. Conclusions

The potential of extracellular vesicles as an alternative way to treat diseases is promising, but it is important to recognize that this field is still in its early stages of development. EVs offer several advantages as drug delivery vehicles, including their natural ability to transport various biomolecules, their potential for targeted delivery to specific cells or tissues, and their ability to minimize adverse effects. However, there are still many challenges that need to be overcome before EV-based therapeutics can be widely adopted in clinical settings. These challenges include issues related to specificity, stability, scalability, and safety. Additionally, there is a need for further research to better understand the mechanisms of EV action, optimize EV isolation and characterization techniques, and evaluate the long-term safety and efficacy of EV-based therapies. Overall, while EVs hold promise as a novel approach to treating diseases, more research and development are needed to fully realize their potential and address current limitations.

## Figures and Tables

**Table 1:** Neurologic effects of the gut microbiome. Selected details of the methods are shown for comparison.

Source Bacteria	Method	Neurologic Effect	Ref.
*Haemophilus influenzae*	In vivo EVs/LPSs Rat model	Induction of blood–brain barrier permeability during experimental meningitis.	[36]
*Helicobacter pylori*	In vivo/in vitro EVs/microglia Mouse model	Induction of neuroinflammation in the CNS, higher prevalence of AD in HP-infected people.	[37]
*Lactobacillus plantarum*	In vivo/in vitro L-EVs/HT22 cells Mouse model	BDNF increase Antidepressant effects in stress-induced depression	[38]
*Lactobacillus plantarum*	In vivo/in vitro L-EVs/neurons	Protection against ischemic brain injury	[39]
*Lactobacillus reuteri*	In vivo Neurons Rat model	Enhancement of excitability of colonic AH neurons	[40]
*Lactobacillus rhamnosus*	In vivo/in vitro/ex vivo EVs/dendritic cells Mouse model	Alteration of nerve-dependent colon migrating motor complexes (MMCs), enteric nerve function, and behavior	[41]
*Paenalcaligenes hominis*	In vivo/in vitro EVs/bacteria/LPSs Mouse model	Risk factor for cognitive decline	[42]

**Table 2:** Neurotransmitters produced by gut bacteria. Data adapted from [63,64].

Dopamine [65,66,67]	Noradrenaline [65,66]	Serotonin [52,66,67,68,69]	Gaba [70,71,72,73,74]	Histamine [67,68,75]
*Escherichia coli*	*Escherichia coli*	*Candida*	*Bifidobacterium adolescentis*	*Citrobacter freuiindii*
*Hafnia alvei*	*Proteus vulgaris*	*Enterococcus*	*Bifidobacterium angulatum*	*Enterobacter* spp.
*Klebsiella pneumoniae*	*Serratia marcescens*	*Escherichia coli*	*Bifidobacterium dentium*	*Hafnia alvei*
*Morganella morganii*		*Hafnia alvei*	*Bifidobacterium infantis*	*Klebsiella pneumoniae*
*Proteus vulgaris*		*Klebsiella grimontii*	*Lactobacillus brevis*	*Lactobacillus lactis*
*Serratia marcescens*		*Klebsiella pneumoniae*	*Lactobacillus paracasei NFRI*	*Lactobacillus plantarum*
		*Lactobacillus lactis* subsp. *Cremoris*	*Lactobacillus plantarum*	*Morganella morganii*
		*Lactobacillus plantarum*	*Lactobacillus reuteri*	*Pediococcus parvulus*
		*Morganella morganii*	*Lactobacillus rhamnosus*	*Streptococcus thermophilus*
			*Streptococcus salivarius*	

**Table 3:** Drugs loaded onto extracellular vesicles.

EV Source	Chemical	Therapeutic Goal	Ref.
Endothelial cells–mimetic nanovesicles	Dapagliflozin	Angiogenesis in diabetic wound healing	[133]
RAW264.7 cells	Linezolid	Antibiotic against *Staphylococcus aureus*	[134]
Pancreatic cancer cells (PCCs), pancreatic stellate cells (PSCs), and macrophages (MØs)	Doxorubicin	Anti-cancer therapy	[135]
Hybrid vector of macrophage-derived microvesicles together with iron oxide nanoparticles	Doxorubicin, tissue-plasminogen activator (t-PA), disulfonated tetraphenyl chlorin-TPCS2a, and 5,10,15,20-tetra(m-hydroxyphenyl) chlorin-mTHPC)	Anti-cancer therapy	[136]
*Klebsiella pneumoniae*	Doxorubicin	Anti-cancer therapy	[137]
Human umbilical vascular endothelial cells (HUVEC)	5,10,15,20-tetra(m-hydroxyphenyl) chlorin-m THPC)	Anti-cancer therapy	[138]
Glioblastoma cells and pancreatic cancer cells (PANC-1)	Paclitaxel	Anti-cancer therapy	[139,140]
Breast cancer cell- and colorectal cancer cell-derived exosomes	Aspirin	Anti-cancer therapy	[141]
HFL-1 (human fetal lung fibroblasts)	Erastin	Anti-cancer therapy	[142]
M2 macrophage-derived exosomes (M2 Exo)	Hexyl 5-aminolevulinate hydrochloride (HAL)	Atherosclerosis treatment	[143]
Allogeneic bone marrow mesenchymal stem cell exosomes (BMSCExo)	Temozolomide	Glioblastoma therapy	[144]
HEI-OC1 cells	Dexamethasone, aspirin, arachidonic, eicosapentaenoic, docosahexaenoic, linoleic acids, lipoxin A4, and resolvin D1	Hearing loss treatment	[145,146]
Stem cells	Curcumin	Regenerative cell therapy	[147]
*E. coli*	Melanin	Cancer monitoring	[148]
*E. coli* and *S. aureus*	Vancomycin and rifampicin	Bacteremia treatment	[149]
*Salmonella typhimurium*	Tegafur	Cancer immunotherapy	[150]
*Enterococcus faecalis*	Capacitabine	Anti-cancer therapy	[151]
*Pseudomonas* *aeruginosa*	Gentamicin	Cepacia syndrome treatment	[152]
*Acinetobacter baumannii*	Levofloxacin	Intestinal bacterial infection treatment	[153]
*E. coli*	Paclitaxel	Anti-cancer therapy	[154]
